# A Rare Case of Juvenile Polyposis Syndrome Mimicking Ménétrier's Disease

**DOI:** 10.7759/cureus.23389

**Published:** 2022-03-22

**Authors:** Michelle Bernshteyn, Abdul Q Bhutta, Jozsef Bordas, Rohin Mehta, Muhammad Osman Arif

**Affiliations:** 1 Internal Medicine, Upstate University Hospital, Syracuse, USA; 2 Gastroenterology, Upstate University Hospital, Syracuse, USA; 3 Pathology, Upstate University Hospital, Syracuse, USA

**Keywords:** colorectal cancer, gi polyposis syndrome, gastrointestinal polyposis, juvenile polyposis syndrome, polyposis, colonoscopy, protein-losing enteropathy, upper extremity deep venous thrombosis, gastroenterology and endoscopy, menetrier's disease

## Abstract

There is a wide differential diagnosis within polyposis syndromes. Our case represents an interesting and diagnostically challenging diagnosis involving a 41-year-old male who presented with an incidental gastric mass on imaging and a colonic mass seen on colonoscopy. Following multiple endoscopic evaluations, histological analysis, and genetic testing, the patient was ultimately diagnosed with juvenile polyposis syndrome (JPS)/hereditary hemorrhagic telangiectasia (HHT) despite the initial suspicion for Ménétrier's disease. His disease course was complicated by an acute upper extremity thrombus and diagnosis of colorectal carcinoma. This case highlights the importance of a thorough evaluation when polyposis syndromes are suspected. Prompt and accurate diagnosis can aid in the treatment, surveillance, and prevention of colorectal carcinoma.

## Introduction

When multiple polyps are visualized during an esophagogastroduodenoscopy (EGD) or colonoscopy, it is important to consider the possibility of an underlying polyposis syndrome. This includes non-hereditary gastrointestinal polyposis, hereditary gastrointestinal polyposis, and hereditary gastrointestinal polyposis cancer syndromes [1]. There is a plethora of polyposis syndromes that differ in disease severity, histological features, and extraintestinal manifestations [2]. Thorough endoscopic evaluation and a multidisciplinary approach involving communication between gastroenterologists, pathologists, and geneticists are warranted to accurately diagnose a polyposis syndrome [3]. Careful family history and awareness of specific extraintestinal symptoms also aid in the diagnosis [4]. Among these syndromes are Ménétrier's disease, a rare protein-losing enteropathy that is underrecognized by clinicians, and juvenile polyposis syndrome (JPS), a devastating autosomal dominant disease. Early and accurate diagnosis has a key role in the treatment and can guide surveillance and prevention of colorectal carcinoma [5].

This article was previously presented as a poster at the 2021 American College of Gastroenterology Annual Scientific Meeting on October 26, 2021.

## Case presentation

**Figure 1:**
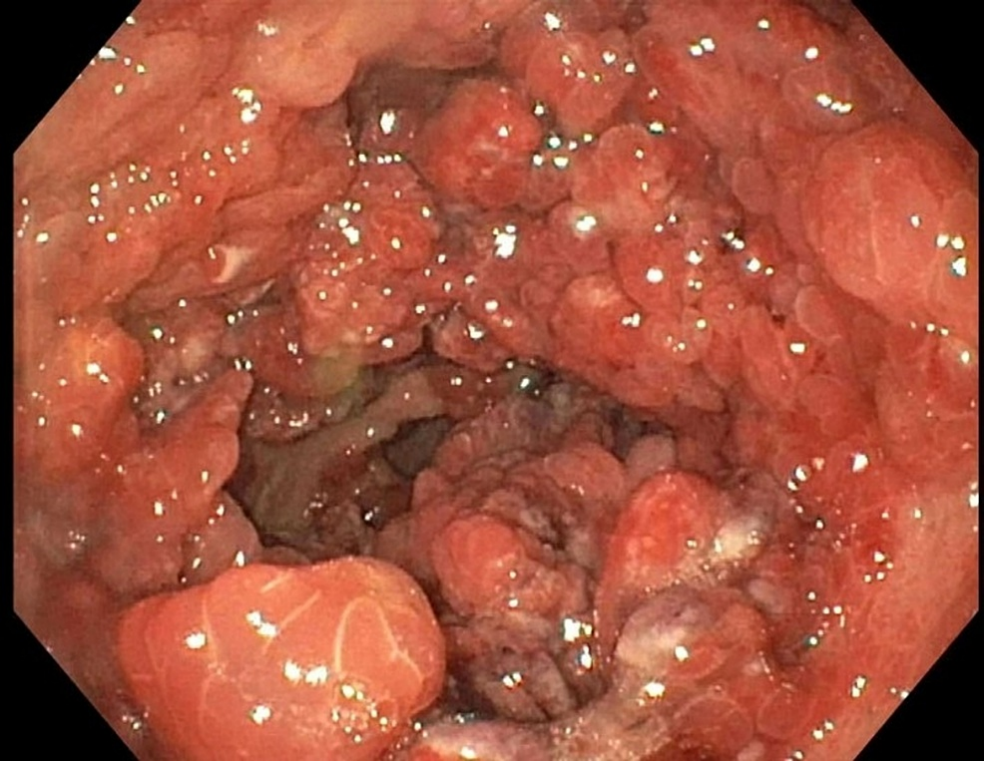
Large fungating and ulcerated masses demonstrated in the gastric body

**Figure 2:**
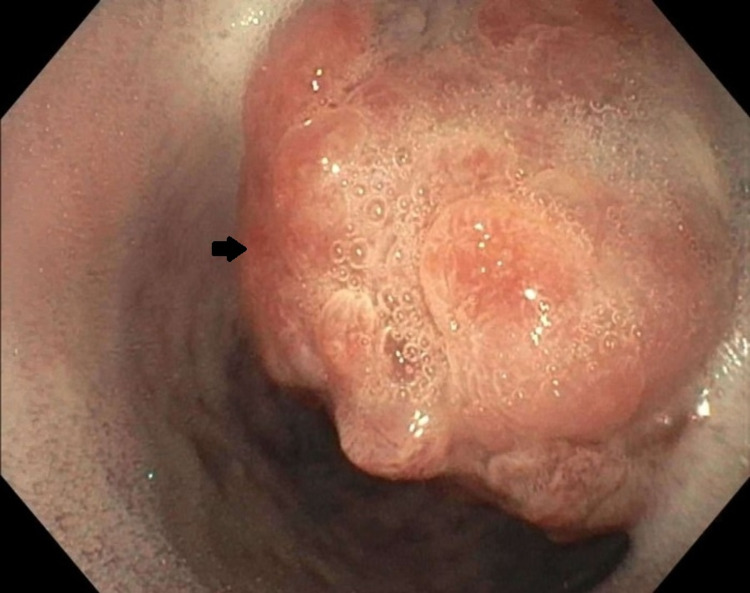
Middle third of the esophagus, prior to cetuximab therapy, demonstrating a mass (arrow)

The patient was a 41-year-old male with a past medical history significant for recently detected fatty liver disease versus cirrhosis with esophageal varices, cerebrovascular accident, mood disorder, hypertension, splenic infarction, and polysubstance use, with an incidental gastric mass on imaging and colonic mass observed on colonoscopy. The patient underwent an EGD, which demonstrated large fungating and ulcerated masses in the stomach and esophagus (Figures 1, 2). Biopsies taken during the EGD were benign and it was determined that deeper biopsies were needed for a definitive diagnosis.

**Figure 3:**
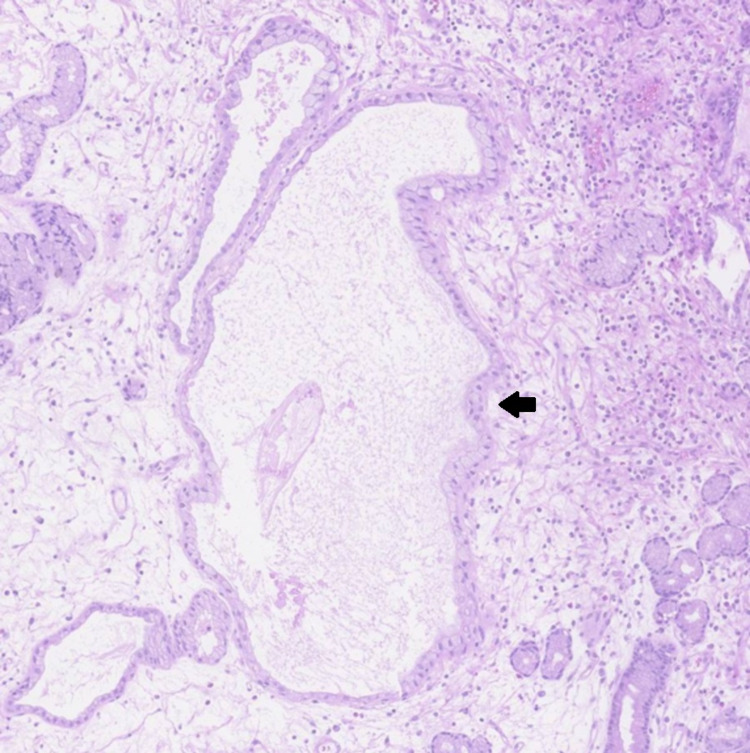
Immunochemical staining of a gastric polyp biopsy demonstrating glandular dilation suggestive of Ménétrier's disease (arrow)

**Figure 4:**
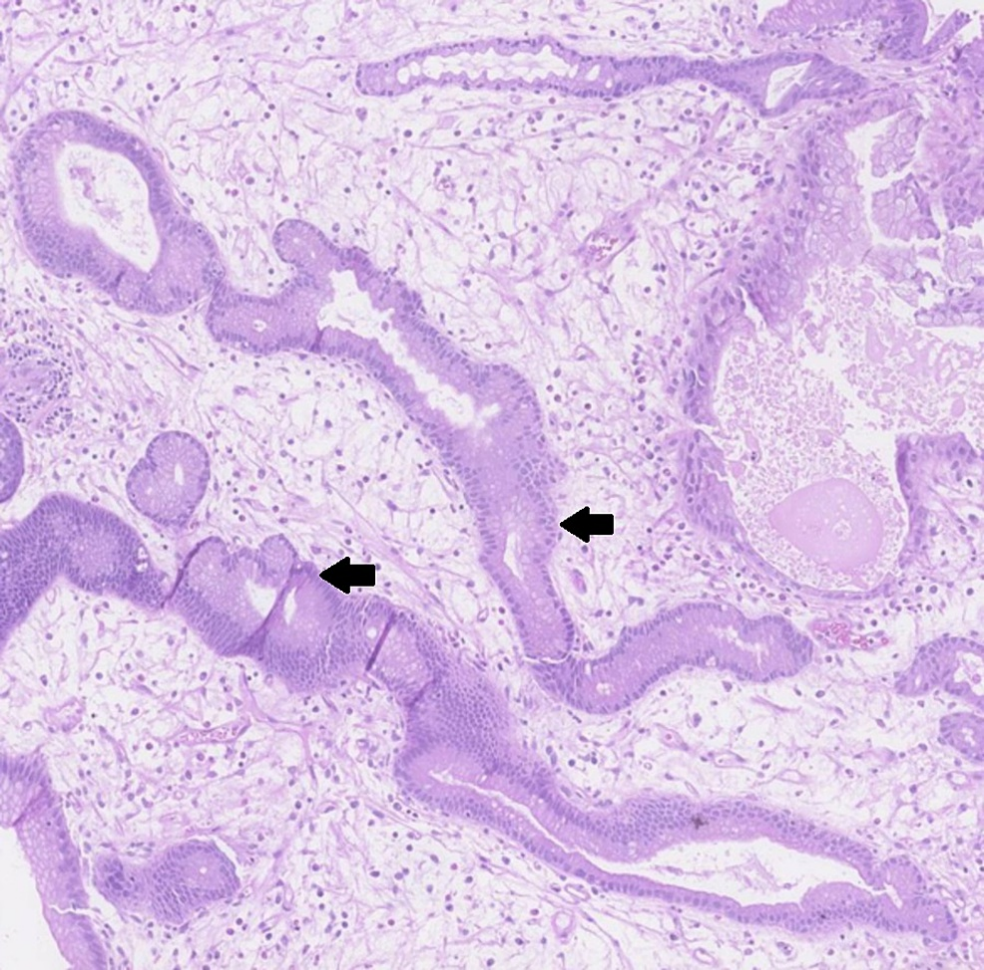
Immunochemical staining of a gastric polyp biopsy demonstrating elongated and tortuous gastric pits suggestive of Ménétrier's disease (arrows)

In the interim, the patient was admitted for hemodynamically insignificant hematemesis. Imaging performed during this hospitalization demonstrated a concern for gastric outlet obstruction. EGD was performed during this encounter and showed new mucosal changes in the duodenum. A biopsy of a gastric polyp was taken, and pathological analysis was suggestive of Ménétrier's disease (Figures 3, 4).

Laboratory studies were notable for elevated chromogranin A at 2,775 ng/mL, calcitonin of 15.5 pg/mL, albumin of 2.1 g/dL, and gastrin of 318 pg/mL. Of note, chromogranin A has a low specificity for carcinoid syndrome. Cytomegalovirus (CMV), serum H. pylori, alpha-1 anti-trypsin, ceruloplasmin, FibroSURE™, HIV, and syphilis testing were negative.

Given the tumor burden and suspicion for Ménétrier's disease, surgery was consulted. After a thorough review of the patient’s case, no surgical intervention was recommended due to the polyp burden. With surgical intervention ruled out, it was decided to treat the patient for Ménétrier's disease with a proton-pump inhibitor twice daily, octreotide 100 mcg four times daily, and cetuximab infusion.

**Figure 5:**
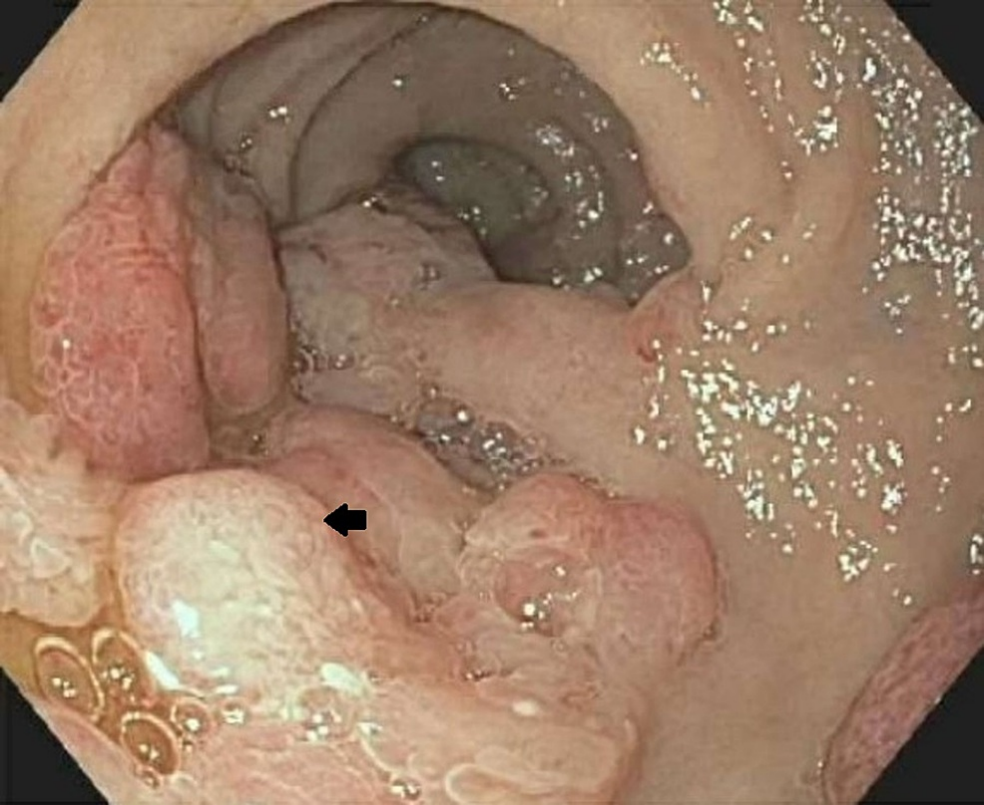
Second portion of the duodenum on follow-up endoscopy demonstrating progression (arrow)

**Figure 6:**
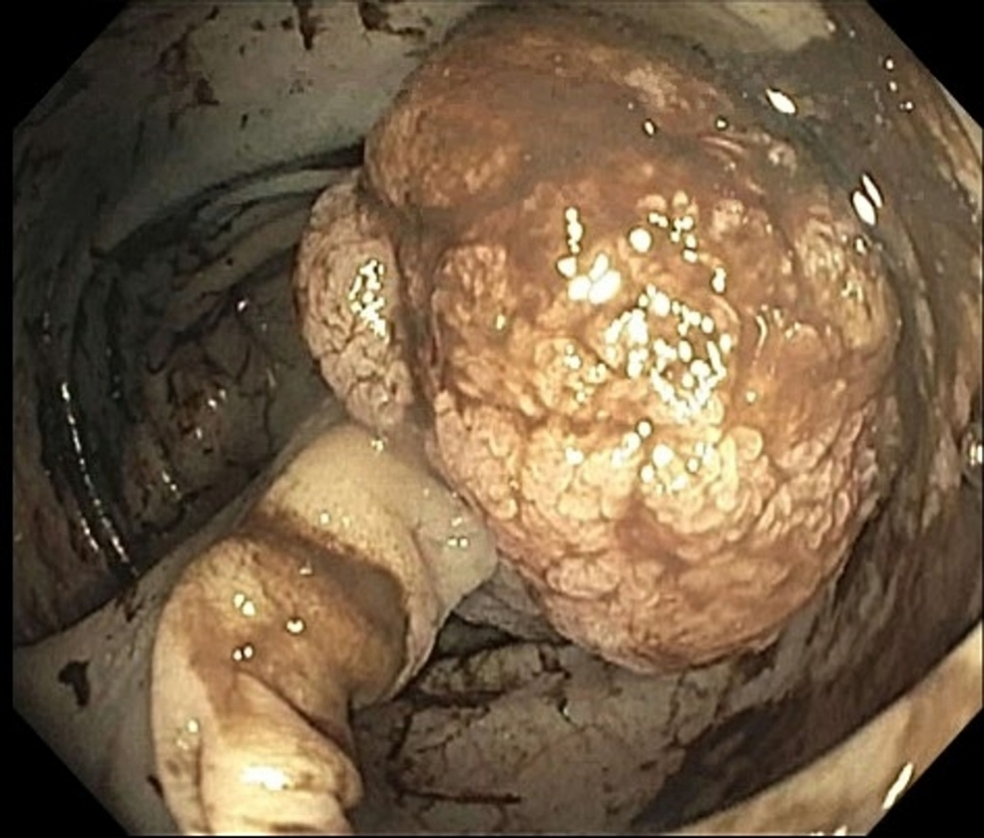
Polyp of the sigmoid colon demonstrated on colonoscopy

**Figure 7:**
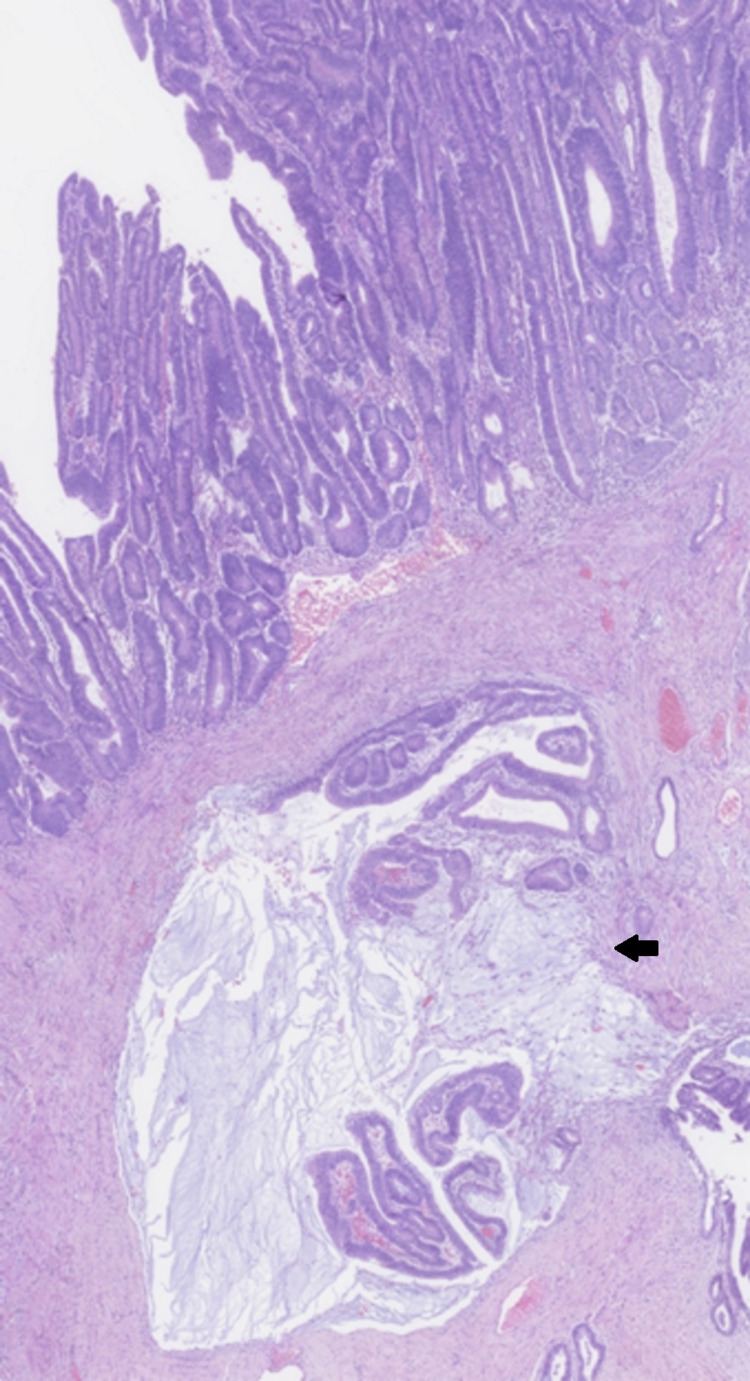
Invasive well-differentiated adenocarcinoma, arising in a tubulovillous adenoma from the sigmoid colon (arrow)

In order to better understand the etiology of the upper gastrointestinal masses seen on EGD and colonic mass seen on a prior colonoscopy, it was recommended that the patient undergo repeat EGD and colonoscopy. However, this was delayed as the patient was found to have an acute upper extremity thrombus and was being treated with anticoagulation. Once deemed appropriate and safe to proceed with the procedures, the patient underwent an EGD, which demonstrated gastric and duodenal polyps but regression of the previously noted esophageal mass (Figure 5). Biopsy of a duodenal polyp revealed a hyperplastic pathology. Gastric pH during this procedure was measured to be acidic at 3.0. Despite poor bowel prep, a sigmoid polyp was identified and removed during the patient’s colonoscopy (Figure 6). It was noted to be suspicious for adenocarcinoma. Pathology confirmed tubulovillous adenoma with extensive high-grade dysplasia and intramucosal carcinoma with focal areas suspicious for adenocarcinoma (Figure 7).

The patient was subsequently started on folinic acid, fluorouracil, and oxaliplatin (FOLFOX) chemotherapy. There was a concern for an underlying hereditary syndrome such as JPS/hereditary hemorrhagic telangiectasia (HHT), Peutz-Jeghers syndrome (PJS), familial adenomatous polyposis (FAP), MUTYH-associated adenomatous polyposis, or possibly gastrointestinal stromal tumor.

He underwent genetic testing, and this was positive for the SMAD4 pathogenic mutation consistent with a diagnosis of JPS/HHT. The patient has since been evaluated by both gastroenterology and surgery who have recommended repeat EGD and colonoscopy, respectively. Unfortunately, the patient declined to undergo the procedures due to unwanted side effects from chemotherapy.

## Discussion

Polyposis syndromes can be classified into multiple categories such as non-hereditary gastrointestinal polyposes including inflammatory and post-inflammatory, hyperplastic, lymphoid, lipomatosis, angiomatosis, leiomyomatosis, pneumatosis cystoids intestinalis, and Cronkhite-Canada Syndrome. Hereditary gastrointestinal polyposis syndromes include FAP, Gardner syndrome, Turcot syndrome, attenuated FAP, hamartomatous polyposes such as PJS, JPS, Cowden syndrome, intestinal ganglioneuromatosis, Bannayan-Riley-Ruvalcaba syndrome, and tuberous sclerosis. Hereditary gastrointestinal polyposis cancer syndromes also include FAP, JPS, and PJS [1,6].

Even though polyposis syndromes cause profound findings on endoscopic evaluation, its diagnosis can be challenging. Polyp number is important along with a histological evaluation. The role of a pathologist is essential as polyps from these syndromes may mimic one another. Deeper biopsies, as was recommended in our case, may be warranted for an adequate sample and better diagnostic yield. It is equally important to obtain a thorough familial history given the strong inheritance pattern of polyposis syndromes [5]. For conditions with similar phenotypes such as FAP and MUTYH-Associated Polyposis (MAP), molecular genetic screening helps to distinguish the diagnosis [4]. Finally, genetic testing has a key role in detecting mutations, which can guide surveillance and colorectal carcinoma prevention for patients and their relatives [5].

Understanding the characteristics of each polyposis syndrome enables a better understanding of the differences in specific cancer risks and complications [5]. Polyposis syndromes are responsible for approximately 1% of all cases of colorectal carcinomas. Moreover, there are associated extracolonic manifestations such as osteomas, hepatoblastomas, extraintestinal tumors, perioral pigmentation, epidermoid cysts, and lipomas [4]. Patients are at risk of protein-loss, gastric-outlet, small-bowel, and esophageal obstruction. Interestingly, there have also been reports of acquired thrombophilia as a direct result [7]. Our patient developed an acute upper extremity deep venous thrombus, which may be linked to his underlying polyposis syndrome. Moreover, he was diagnosed with adenocarcinoma. Thus, it is important for clinicians to be aware of these diseases.

Ménétrier's is a rare disease caused by increased transdermal growth factor-α expression and heightened epidermal growth factor receptor (EGFR) activity [8]. It can present with hematemesis [9]. It is a protein-losing gastroenteropathy with severe thickening of the gastric mucosa. Histological features include gland dilation and gastric pit expansion. Labs show elevated gastrin and low albumin. CMV often coexists with the condition in the pediatric population [10]. While octreotide can control EGFR signaling, cetuximab has been demonstrated to cause remission [11].

JPS is an autosomal dominant disease, which typically presents in the third decade of life; it has been reported to have a 20-70% risk of colorectal carcinoma and a 20% risk of gastric cancer. Patients with five or more juvenile polyps within the colon or any juvenile polyps in the rest of the gastrointestinal tract should undergo evaluation for this condition [12]. Genetic testing for SMAD4 and BMPR1A can assist with the diagnosis, as in our patient [12]. Given the high risk for malignancy, diagnostic recommendations include evaluation of the upper gastrointestinal tract with endoscopy to assess for stomach and small bowel involvement, undergoing polypectomies every one to three years, and consideration of a colectomy versus proctocolectomy [1,5]. It is also important for the patients' family members to be screened.

When diagnosing JPS, the role of the pathologist is essential. Some polyps may mimic inflammatory polyps and are indistinguishable from pseudopolyps seen in inflammatory bowel diseases. JPS polyps have dilated crypts filled with neutrophils and are surrounded by inflamed stroma [3]. Suspicion occurs if a patient has five or more colorectal juvenile polyps, in case of any colorectal juvenile polyps in a patient with a positive family history, or multiple polyps in both the upper and lower gastrointestinal tracts [5].

In our patient, there was a concern for an underlying hereditary syndrome such as PJS, FAP, and MAP. PJS should be suspected in patients who demonstrate perioral or buccal hyper-pigmentation, family history of PJS, or two or more hamartomatous polyps [12]. These patients should undergo STK11 genetic testing and, if positive, are recommended to undergo screening for colorectal, gastric, small bowel, pancreatic, breast, ovarian, cervical, uterine, and testicular cancer. Screening should occur around the second decade of life [12]. FAP should be suspected in patients with more than 10 colorectal adenomas or strong family history for FAP. Genetic testing should include APC and MUTYH. Annual colonoscopy or flexible sigmoidoscopy and consideration of colectomy should occur starting at puberty [12]. These patients should be screened for proximal small bowel and gastric cancer between the ages of 25-30 years. They are also recommended to undergo annual ultrasound of the thyroid to assess for nodules [12]. On the other hand, if they are positive for the MUTYH mutation, this would confirm the presence of MAP. These patients are recommended to undergo colonoscopy every one to two years starting between the ages of 25-30 years as well as a baseline upper endoscopy between the ages of 30-35 years.

## Conclusions

This case report highlights the complexities in the diagnosis of polyposis syndromes. Ménétrier's disease and JPS/HHT are two important differential diagnoses to consider. Moreover, we have highlighted many complications that can be associated with these diseases. Polyposis syndrome patients are at a high risk of colorectal carcinoma and extracolonic manifestations. Early detection is essential for patients and their relatives. Even though initial suspicion is based on endoscopic findings, a multidisciplinary approach towards polyp histology and genetic testing is warranted.
